# Crystal structure of 2-amino-3-cyano-4-(4-meth­oxy­phen­yl)-4*H*-1-benzo­thieno[3,2-*b*]pyran

**DOI:** 10.1107/S2056989015023464

**Published:** 2015-12-12

**Authors:** Mohamed Bakhouch, Mohamed El Yazidi, Abdelali Kerbal, Mohamed Saadi, Lahcen El Ammari

**Affiliations:** a Laboratoire de Chimie Organique, Faculté des Sciences Dhar el Mahraz, Université Sidi Mohammed Ben Abdellah, BP 1796 Atlas, 30000 Fès, Morocco; b Laboratoire de Chimie du Solide Appliquée, Faculté des Sciences, Université Mohammed V de Rabat, Avenue Ibn Battouta, BP 1014, Rabat, Morocco

**Keywords:** crystal structure, benzothino[3,2-*b*]pyran, 2-amino-4-aryl-4*H*-pyran

## Abstract

The three fused five- and six-membered rings in the title compound, C_19_H_14_N_2_O_2_S, are virtually coplanar, with the maximum deviation from the mean plane being 0.060 (1) Å. This benzothieno[3,2-*b*]pyran ring system is nearly perpendic­ular to the plane of the 4-meth­oxy­phenyl ring, forming a dihedral angle of 83.65 (5)°. In the crystal, mol­ecules are linked by pairs of N—H⋯N hydrogen bonds into inversion dimers. The dimeric units are further connected by an N—H⋯O hydrogen bond into a tape running along the *b* axis. The tapes are linked together by C—H⋯N and π–π inter­actions [centroid–centroid distance = 3.7743 (8) Å], forming a three-dimensional network.

## Related literature   

For biological properties of 2-amino-4-aryl-4*H*-pyran derivatives, see: Panda *et al.* (1997 ▸); Mungra *et al.* (2011 ▸). For the reactivity of (*Z*)-2-aryl­idenebenzo[*b*]thio­phen-3(2*H*)-ones (thio­aurones), see: Boughaleb *et al.* (2010 ▸, 2011 ▸); Bakhouch *et al.* (2015 ▸). For a related structure, see: Bakhouch *et al.* (2014 ▸).
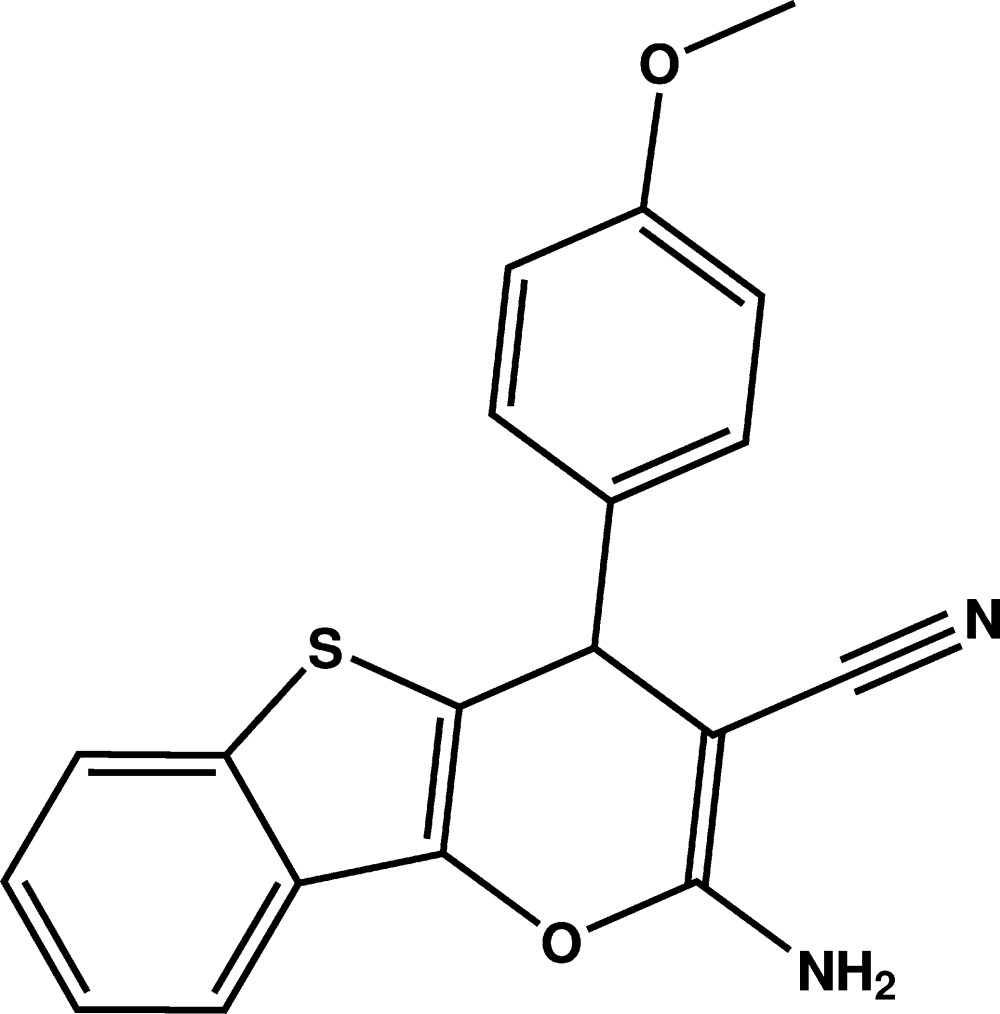


## Experimental   

### Crystal data  

C_19_H_14_N_2_O_2_S
*M* *_r_* = 334.38
Triclinic, 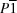

*a* = 6.0469 (3) Å
*b* = 10.8135 (5) Å
*c* = 13.3260 (6) Å
α = 109.943 (2)°
β = 93.226 (2)°
γ = 95.439 (2)°
*V* = 811.76 (7) Å^3^
*Z* = 2
Mo *K*α radiation
μ = 0.21 mm^−1^
*T* = 296 K
0.40 × 0.37 × 0.24 mm

### Data collection  

Bruker X8 APEX diffractometer
33610 measured reflections
4563 independent reflections
3683 reflections with *I* > 2σ(*I*)
*R*_int_ = 0.030

### Refinement  

*R*[*F* ^2^ > 2σ(*F* ^2^)] = 0.039
*wR*(*F* ^2^) = 0.123
*S* = 1.02
4563 reflections
217 parameters
H-atom parameters constrained
Δρ_max_ = 0.30 e Å^−3^
Δρ_min_ = −0.26 e Å^−3^

### 

Data collection: *APEX2* (Bruker, 2009 ▸); cell refinement: *SAINT* (Bruker, 2009 ▸); data reduction: *SAINT*; program(s) used to solve structure: *SHELXT* (Sheldrick, 2015*a* ▸); program(s) used to refine structure: *SHELXL2014* (Sheldrick, 2015*b* ▸); molecular graphics: *ORTEP-3 for Windows* (Farrugia, 2012 ▸); software used to prepare material for publication: *PLATON* (Spek, 2009 ▸) and *publCIF* (Westrip, 2010 ▸).

## Supplementary Material

Crystal structure: contains datablock(s) I. DOI: 10.1107/S2056989015023464/is5436sup1.cif

Structure factors: contains datablock(s) I. DOI: 10.1107/S2056989015023464/is5436Isup2.hkl

Supporting information file. DOI: 10.1107/S2056989015023464/is5436Isup3.cml

**Fig. 1.**
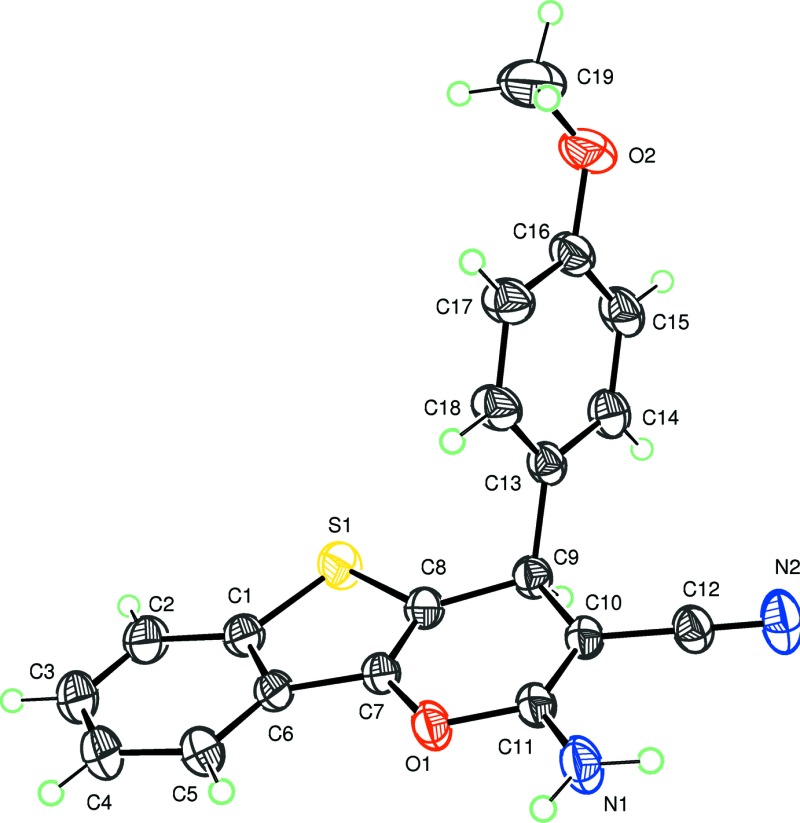
The molecular structure of the title compound with the atom-labelling scheme. Displacement ellipsoids are drawn at the 50% probability level. H atoms are represented as small circles.

. DOI: 10.1107/S2056989015023464/is5436fig1.tif
The mol­ecular structure of the title compound with the atom-labelling scheme. Displacement ellipsoids are drawn at the 50% probability level. H atoms are represented as small circles.

**Fig. 2.**
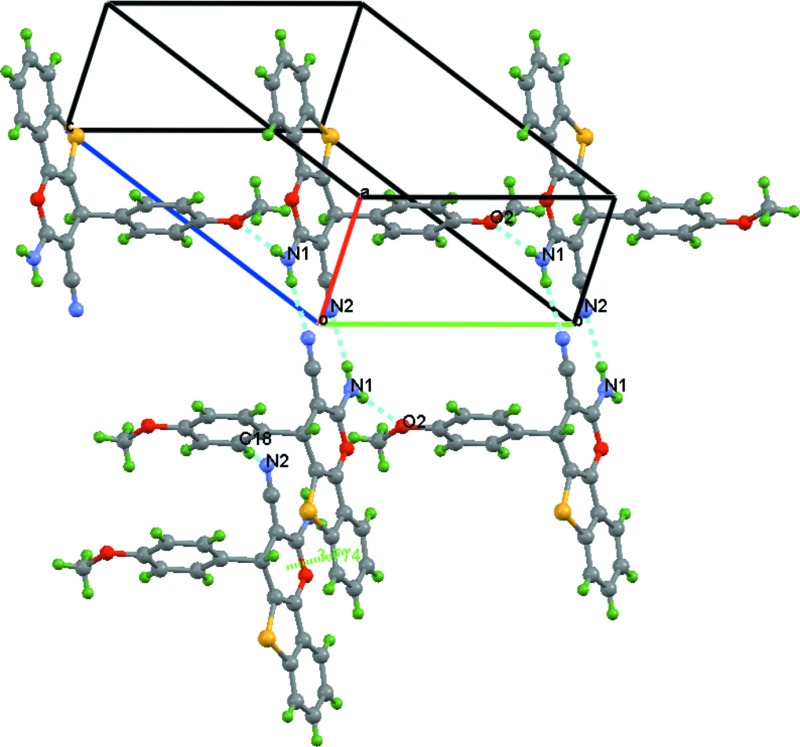
A packing diagram of the title compound showing molecules linked by hydrogen bonds (dashed blue lines) and a π–π interaction (dashed green line).

. DOI: 10.1107/S2056989015023464/is5436fig2.tif
A packing diagram of the title compound showing mol­ecules linked by hydrogen bonds (dashed blue lines) and a π–π inter­action (dashed green line).

CCDC reference: 1440832

Additional supporting information:  crystallographic information; 3D view; checkCIF report

## Figures and Tables

**Table 1:** Hydrogen-bond geometry (Å, °)

*D*—H⋯*A*	*D*—H	H⋯*A*	*D*⋯*A*	*D*—H⋯*A*
N1—H1*A*⋯N2^i^	0.86	2.18	3.0060 (17)	160
N1—H1*B*⋯O2^ii^	0.86	2.19	3.0158 (16)	162
C18—H18⋯N2^iii^	0.93	2.49	3.3997 (17)	165

Symmetry codes: (i) 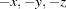; (ii) 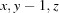; (iii) 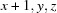.

## supplementary crystallographic information

### S1. Comment

Substituted 2-amino-4-aryl-4*H*-pyran derivatives are an important class of heterocyclic compounds, which frequently exhibit a wide range of biological properties *viz*. antiproliferative and antitubercular activities (Panda *et al.*, 1997; Mungra *et al.*, 2011). Thus, in view of the large spectrum of application of these compounds and in continuation of ongoing research focused on the reactivity of the (*Z*)-2-arylidenebenzo[*b*]thiophen-3(2*H*)-ones (thioaurones) (Boughaleb *et al.*, 2010, 2011; Bakhouch *et al.*, 2015), we describe herein the behavior of ethyl cyanoacetate with (*Z*)-2-(4-methoxybenzylidene)benzo[*b*]thiophen-3(2*H*)-one (Bakhouch *et al.*, 2014). Initially the condensation furnish the Michael adducts, which undergoes intramolecular cyclization to afford imino-pyran. The subsequent tautomeric transformation gives rise to 2-amino-3-cyano-4-(4-methoxyphenyl)-4*H*-1-benzothieno[3,2-*b*]pyran.

The title molecule is formed by a benzothieno[3,2-*b*]pyran system linked to the 4-methoxyphenyl ring as shown in Fig. 1. The three fused rings are almost coplanar with a maximum deviation from the mean plane being 0.060 (1) Å at C9. The dihedral angle between the benzothieno[3,2-*b*]pyran ring system and the mean plane of the 4-methoxyphenyl ring is 83.65 (5)°. In the crystal, molecules are linked by pairs of N—H···N hydrogen bonds into centrosymmetric dimeric units, which are further connected by N—H···O interactions to build tapes along the *b* axis (Table 1). The tapes are linked together by C—H···N and π–π interactions [centroid-centroid distance = 3.7743 (8) Å] to form a three-dimensional network as shown in Fig. 2.

### S2. Experimental

In a 100 ml flask equipped with a condenser was dissolved 4 mmol of (*Z*)-2-(4-methoxybenzylidene)-1-benzo[*b*]thiophen-3(2*H*)-one and 5 mmol of malononitrile in 30 ml of ethanol. Then, 1 ml of piperidine was added, and the reaction mixture was refluxed for 6 h. Thin layer chromatography revealed the formation of a single product. The organic phase was evaporated under reduce pressure. The resulting residue was recrystallized from ethanol (yield 77%; *m.p.* 515 K). Single crystals of the title compound suitable for X-ray diffraction were obtained from slow evaporation of an ethanol solution.

### S3. Refinement

H atoms were located in a difference map and treated as riding with C—H = 0.96, 0.98 and 0.93 Å for methyl, methine and aromatic, respectively, and N—H = 0.86 Å. The *U*_iso_(H) values were set at 1.2*U*_eq_(C, N) for methine, aromatic and N—H or 1.5*U*_eq_(C) for methyl. The reflection (0 0 1) affected by the beamstop was removed during refinement.

### Crystal data

**Table:**

C_19_H_14_N_2_O_2_S	*F*(000) = 348
*M**_r_* = 334.38	*D*_x_ = 1.368 Mg m^−^^3^
Triclinic, *P*1	Melting point: 515 K
*a* = 6.0469 (3) Å	Mo *K*α radiation, λ = 0.71073 Å
*b* = 10.8135 (5) Å	Cell parameters from 4563 reflections
*c* = 13.3260 (6) Å	θ = 2.1–29.6°
α = 109.943 (2)°	µ = 0.21 mm^−^^1^
β = 93.226 (2)°	*T* = 296 K
γ = 95.439 (2)°	Block, colourless
*V* = 811.76 (7) Å^3^	0.40 × 0.37 × 0.24 mm
*Z* = 2	

### Data collection

**Table:**

Bruker X8 APEX diffractometer	3683 reflections with *I* > 2σ(*I*)
Radiation source: fine-focus sealed tube	*R*_int_ = 0.030
Graphite monochromator	θ_max_ = 29.6°, θ_min_ = 2.1°
φ and ω scans	*h* = −8→8
33610 measured reflections	*k* = −15→15
4563 independent reflections	*l* = −18→18

### Refinement

**Table:**

Refinement on *F*^2^	0 restraints
Least-squares matrix: full	Hydrogen site location: inferred from neighbouring sites
*R*[*F*^2^ > 2σ(*F*^2^)] = 0.039	H-atom parameters constrained
*wR*(*F*^2^) = 0.123	*w* = 1/[σ^2^(*F*_o_^2^) + (0.0643*P*)^2^ + 0.197*P*] where *P* = (*F*_o_^2^ + 2*F*_c_^2^)/3
*S* = 1.02	(Δ/σ)_max_ < 0.001
4563 reflections	Δρ_max_ = 0.30 e Å^−^^3^
217 parameters	Δρ_min_ = −0.26 e Å^−^^3^

### Special details

**Table:**

Geometry. All e.s.d.'s (except the e.s.d. in the dihedral angle between two l.s. planes) are estimated using the full covariance matrix. The cell e.s.d.'s are taken into account individually in the estimation of e.s.d.'s in distances, angles and torsion angles; correlations between e.s.d.'s in cell parameters are only used when they are defined by crystal symmetry. An approximate (isotropic) treatment of cell e.s.d.'s is used for estimating e.s.d.'s involving l.s. planes.

### Fractional atomic coordinates and isotropic or equivalent isotropic displacement parameters (Å^2^)

**Table:**

	*x*	*y*	*z*	*U*_iso_*/*U*_eq_	
C1	0.9654 (2)	0.25649 (14)	0.46217 (11)	0.0435 (3)	
C2	1.1515 (3)	0.27892 (17)	0.53633 (13)	0.0560 (4)	
H2	1.1870	0.3600	0.5917	0.067*	
C3	1.2798 (3)	0.17813 (19)	0.52500 (15)	0.0630 (5)	
H3	1.4041	0.1914	0.5735	0.076*	
C4	1.2285 (3)	0.05642 (19)	0.44279 (15)	0.0592 (4)	
H4	1.3203	−0.0096	0.4365	0.071*	
C5	1.0432 (2)	0.03195 (16)	0.37019 (12)	0.0481 (3)	
H5	1.0082	−0.0501	0.3159	0.058*	
C6	0.9100 (2)	0.13312 (13)	0.38033 (10)	0.0387 (3)	
C7	0.7137 (2)	0.13537 (12)	0.31555 (10)	0.0361 (3)	
C8	0.6294 (2)	0.25117 (12)	0.34268 (10)	0.0368 (3)	
C9	0.4291 (2)	0.27703 (12)	0.28465 (10)	0.0354 (2)	
H9	0.3147	0.3014	0.3347	0.042*	
C10	0.3425 (2)	0.14569 (12)	0.19663 (10)	0.0351 (2)	
C11	0.4390 (2)	0.03198 (12)	0.17479 (10)	0.0376 (3)	
C12	0.1396 (2)	0.14023 (12)	0.13670 (10)	0.0389 (3)	
C13	0.4803 (2)	0.38855 (11)	0.24049 (9)	0.0347 (2)	
C14	0.3445 (2)	0.48861 (13)	0.25600 (11)	0.0425 (3)	
H14	0.2199	0.4878	0.2937	0.051*	
C15	0.3923 (3)	0.58966 (14)	0.21602 (13)	0.0483 (3)	
H15	0.3001	0.6563	0.2272	0.058*	
C16	0.5767 (2)	0.59197 (13)	0.15942 (11)	0.0435 (3)	
C17	0.7125 (3)	0.49179 (15)	0.14233 (13)	0.0496 (3)	
H17	0.8362	0.4919	0.1040	0.060*	
C18	0.6619 (2)	0.39158 (14)	0.18300 (12)	0.0463 (3)	
H18	0.7531	0.3244	0.1712	0.056*	
C19	0.8083 (4)	0.7080 (2)	0.0723 (2)	0.0811 (6)	
H19A	0.8128	0.7853	0.0522	0.122*	
H19B	0.9378	0.7155	0.1201	0.122*	
H19C	0.8065	0.6307	0.0094	0.122*	
N1	0.3666 (2)	−0.08499 (12)	0.09804 (11)	0.0568 (4)	
H1A	0.2477	−0.0932	0.0567	0.068*	
H1B	0.4392	−0.1518	0.0902	0.068*	
N2	−0.0263 (2)	0.13768 (13)	0.08954 (11)	0.0548 (3)	
O1	0.62572 (16)	0.02236 (9)	0.23233 (8)	0.0432 (2)	
O2	0.6139 (2)	0.69683 (11)	0.12427 (11)	0.0626 (3)	
S1	0.78335 (7)	0.36908 (4)	0.45395 (3)	0.04948 (12)	

### Atomic displacement parameters (Å^2^)

**Table:**

	*U*^11^	*U*^22^	*U*^33^	*U*^12^	*U*^13^	*U*^23^
C1	0.0426 (7)	0.0494 (7)	0.0399 (6)	−0.0059 (5)	−0.0089 (5)	0.0224 (6)
C2	0.0547 (9)	0.0610 (9)	0.0499 (8)	−0.0129 (7)	−0.0208 (7)	0.0254 (7)
C3	0.0489 (9)	0.0776 (11)	0.0678 (10)	−0.0069 (8)	−0.0239 (8)	0.0405 (9)
C4	0.0463 (8)	0.0718 (11)	0.0686 (10)	0.0073 (7)	−0.0116 (7)	0.0387 (9)
C5	0.0443 (7)	0.0536 (8)	0.0508 (8)	0.0039 (6)	−0.0070 (6)	0.0263 (6)
C6	0.0351 (6)	0.0469 (7)	0.0384 (6)	−0.0032 (5)	−0.0055 (5)	0.0238 (5)
C7	0.0352 (6)	0.0383 (6)	0.0349 (6)	−0.0018 (5)	−0.0060 (5)	0.0159 (5)
C8	0.0381 (6)	0.0372 (6)	0.0333 (5)	−0.0011 (5)	−0.0055 (5)	0.0129 (5)
C9	0.0353 (6)	0.0352 (6)	0.0342 (6)	0.0051 (4)	−0.0008 (4)	0.0106 (5)
C10	0.0335 (6)	0.0351 (6)	0.0358 (6)	0.0025 (4)	−0.0042 (4)	0.0127 (5)
C11	0.0367 (6)	0.0351 (6)	0.0403 (6)	0.0013 (5)	−0.0084 (5)	0.0148 (5)
C12	0.0393 (6)	0.0357 (6)	0.0385 (6)	0.0094 (5)	−0.0026 (5)	0.0087 (5)
C13	0.0365 (6)	0.0308 (5)	0.0341 (6)	0.0070 (4)	−0.0023 (4)	0.0080 (4)
C14	0.0394 (6)	0.0439 (7)	0.0437 (7)	0.0148 (5)	0.0046 (5)	0.0121 (5)
C15	0.0497 (8)	0.0405 (7)	0.0572 (8)	0.0210 (6)	0.0018 (6)	0.0166 (6)
C16	0.0502 (7)	0.0343 (6)	0.0475 (7)	0.0082 (5)	−0.0039 (6)	0.0165 (5)
C17	0.0496 (8)	0.0461 (7)	0.0617 (9)	0.0162 (6)	0.0167 (7)	0.0252 (7)
C18	0.0472 (7)	0.0388 (6)	0.0604 (8)	0.0204 (6)	0.0147 (6)	0.0215 (6)
C19	0.0820 (14)	0.0726 (12)	0.1117 (17)	0.0095 (10)	0.0202 (13)	0.0596 (13)
N1	0.0588 (8)	0.0342 (6)	0.0648 (8)	0.0082 (5)	−0.0296 (6)	0.0061 (5)
N2	0.0480 (7)	0.0517 (7)	0.0530 (7)	0.0193 (5)	−0.0130 (5)	0.0027 (6)
O1	0.0421 (5)	0.0351 (4)	0.0482 (5)	0.0055 (4)	−0.0155 (4)	0.0119 (4)
O2	0.0705 (8)	0.0488 (6)	0.0832 (8)	0.0155 (5)	0.0089 (6)	0.0395 (6)
S1	0.0563 (2)	0.04306 (19)	0.04101 (19)	−0.00067 (15)	−0.01393 (15)	0.00898 (14)

### Geometric parameters (Å, º)

**Table:**

C1—C2	1.4007 (19)	C11—N1	1.3416 (16)
C1—C6	1.4011 (19)	C11—O1	1.3612 (14)
C1—S1	1.7427 (15)	C12—N2	1.1462 (17)
C2—C3	1.369 (3)	C13—C18	1.3779 (19)
C2—H2	0.9300	C13—C14	1.3867 (17)
C3—C4	1.389 (3)	C14—C15	1.384 (2)
C3—H3	0.9300	C14—H14	0.9300
C4—C5	1.382 (2)	C15—C16	1.384 (2)
C4—H4	0.9300	C15—H15	0.9300
C5—C6	1.394 (2)	C16—O2	1.3717 (16)
C5—H5	0.9300	C16—C17	1.3858 (19)
C6—C7	1.4349 (16)	C17—C18	1.385 (2)
C7—C8	1.3385 (18)	C17—H17	0.9300
C7—O1	1.3755 (14)	C18—H18	0.9300
C8—C9	1.4997 (17)	C19—O2	1.414 (2)
C8—S1	1.7382 (12)	C19—H19A	0.9600
C9—C10	1.5233 (16)	C19—H19B	0.9600
C9—C13	1.5265 (17)	C19—H19C	0.9600
C9—H9	0.9800	N1—H1A	0.8600
C10—C11	1.3594 (17)	N1—H1B	0.8600
C10—C12	1.4110 (16)		
			
C2—C1—C6	120.66 (14)	N1—C11—O1	110.43 (11)
C2—C1—S1	127.61 (13)	C10—C11—O1	122.91 (11)
C6—C1—S1	111.71 (10)	N2—C12—C10	178.75 (15)
C3—C2—C1	118.14 (15)	C18—C13—C14	118.18 (12)
C3—C2—H2	120.9	C18—C13—C9	120.75 (11)
C1—C2—H2	120.9	C14—C13—C9	121.07 (12)
C2—C3—C4	121.54 (14)	C15—C14—C13	120.76 (13)
C2—C3—H3	119.2	C15—C14—H14	119.6
C4—C3—H3	119.2	C13—C14—H14	119.6
C5—C4—C3	121.02 (16)	C16—C15—C14	120.31 (12)
C5—C4—H4	119.5	C16—C15—H15	119.8
C3—C4—H4	119.5	C14—C15—H15	119.8
C4—C5—C6	118.42 (15)	O2—C16—C15	116.47 (12)
C4—C5—H5	120.8	O2—C16—C17	124.00 (14)
C6—C5—H5	120.8	C15—C16—C17	119.52 (13)
C5—C6—C1	120.21 (12)	C18—C17—C16	119.31 (13)
C5—C6—C7	129.87 (13)	C18—C17—H17	120.3
C1—C6—C7	109.91 (12)	C16—C17—H17	120.3
C8—C7—O1	124.87 (11)	C13—C18—C17	121.91 (12)
C8—C7—C6	115.62 (11)	C13—C18—H18	119.0
O1—C7—C6	119.51 (11)	C17—C18—H18	119.0
C7—C8—C9	124.73 (11)	O2—C19—H19A	109.5
C7—C8—S1	111.22 (9)	O2—C19—H19B	109.5
C9—C8—S1	124.05 (9)	H19A—C19—H19B	109.5
C8—C9—C10	105.99 (10)	O2—C19—H19C	109.5
C8—C9—C13	113.02 (10)	H19A—C19—H19C	109.5
C10—C9—C13	112.31 (10)	H19B—C19—H19C	109.5
C8—C9—H9	108.5	C11—N1—H1A	120.0
C10—C9—H9	108.5	C11—N1—H1B	120.0
C13—C9—H9	108.5	H1A—N1—H1B	120.0
C11—C10—C12	117.49 (11)	C11—O1—C7	116.13 (10)
C11—C10—C9	125.31 (11)	C16—O2—C19	118.42 (13)
C12—C10—C9	117.12 (10)	C8—S1—C1	91.51 (6)
N1—C11—C10	126.65 (12)		

### Hydrogen-bond geometry (Å, º)

**Table:**

*D*—H···*A*	*D*—H	H···*A*	*D*···*A*	*D*—H···*A*
N1—H1*A*···N2^i^	0.86	2.18	3.0060 (17)	160
N1—H1*B*···O2^ii^	0.86	2.19	3.0158 (16)	162
C18—H18···N2^iii^	0.93	2.49	3.3997 (17)	165

Symmetry codes: (i) −*x*, −*y*, −*z*; (ii) *x*, *y*−1, *z*; (iii) *x*+1, *y*, *z*.
