# Supplementary figures and images for: Crystal structure of 2-amino-3-cyano-4-(4-meth­oxy­phen­yl)-4H-1-benzo­thieno[3,2-b]pyran
Source: Acta Crystallogr E Crystallogr Commun. 2015 Dec 12;71(Pt 12):o1043–4. doi: 10.1107/S2056989015023464 (PMC4719970; doi:10.1107/S2056989015023464)

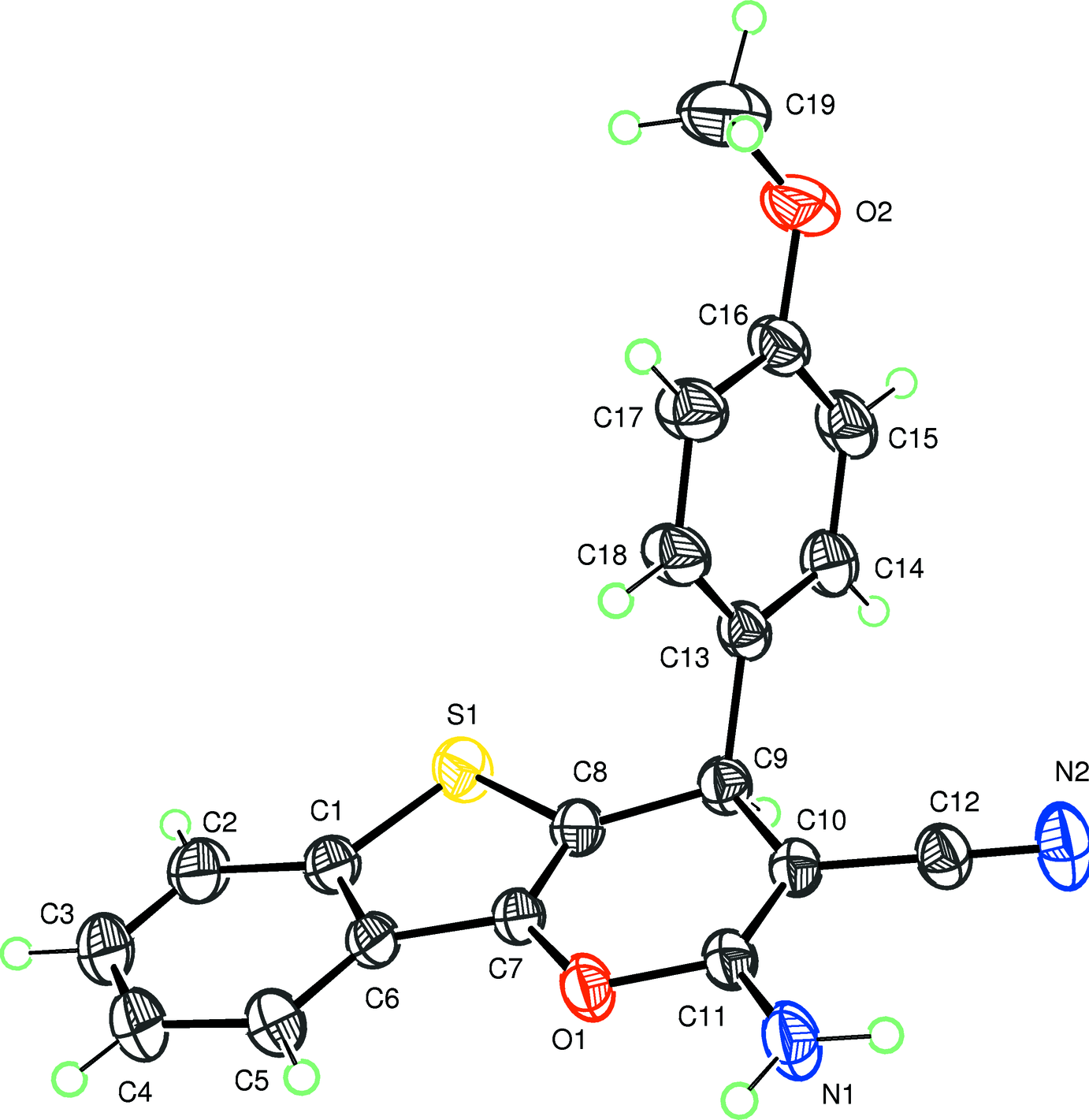

Supplement: Supplementary file 4 [file e-71-o1043-fig1.tif]

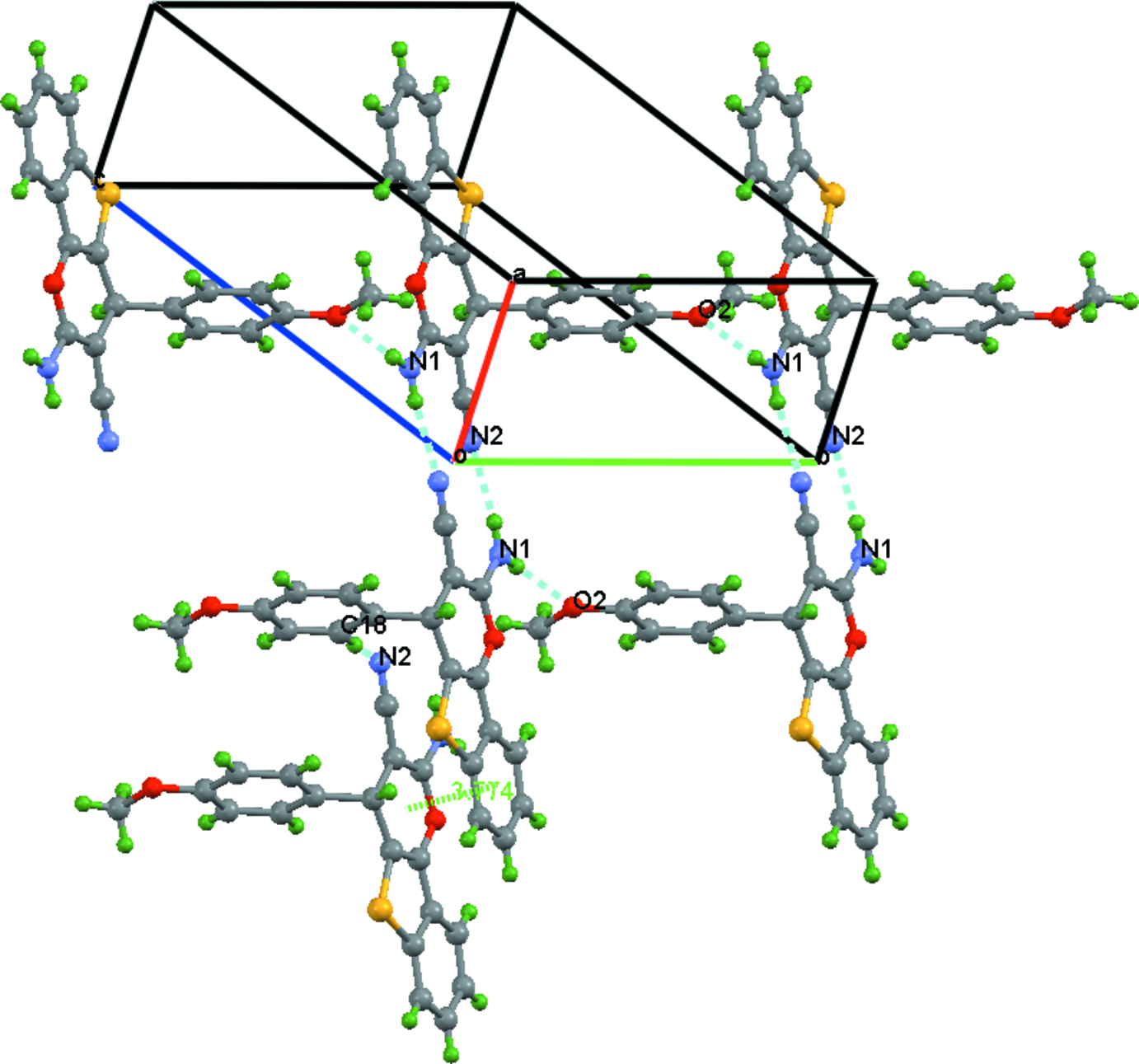

Supplement: Supplementary file 5 [file e-71-o1043-fig2.tif]
